
JOURNAL INFORMATION
==============================
NLM Title Abbreviation: Behav Neurol
Journal ID: Behav Neurol
NLM Journal ID: 8914585
Journal ID: BN

Behavioural Neurology

ISSN: 0953-4180
EISSN: 1875-8584
Publisher: Wiley

ARTICLE INFORMATION
==============================
PMCID: PMC5434414
PMID: 21422545
DOI: 10.3233/BEN-2010-0285
Article ID: 925328
Article version: 1
Subjects: Research Article

An Estimate of Lifetime Cognitive Change and Its Relationship with Diabetes Health in Older Adults with Type 1 Diabetes: Preliminary Results

Johnston Harriet 1 *
McCrimmon Rory 2
Petrie John 3
Astell Arlene 1
1 University of St. Andrews St. Andrews UK
2 University of Dundee Dundee UK
3 BHF Glasgow Cardiovascular Research Centre Glasgow UK
*Harriet Johnston: hnj2@st-andrews.ac.uk
Print publication date: 2010
Electronic publication date: 2011 Mar 7
Volume: 23
Issue: 4
First page: 165
Last page: 167
Received 2011 Jan 31; Accepted 2011 Jan 31
Copyright: Copyright © 2010 Hindawi Publishing Corporation and the authors.
Copyright year: 2010
License: This is an open access article distributed under the Creative Commons Attribution License, which permits unrestricted use, distribution, and reproduction in any medium, provided the original work is properly cited.
License URL: https://creativecommons.org/licenses/by/3.0/

==============================
Behavioural Neurology 23 (2010) 165–167
DOI 10.3233/BEN-2010-0285
IOS Press

### An estimate of lifetime cognitive change and its relationship with diabetes health in older adults with type 1 diabetes: Preliminary results

Harriet Johnston[a,*], Rory McCrimmon[b], John Petrie[c] and Arlene Astell[a]
[a] *University of St. Andrews, St. Andrews, UK*
[b] *University of Dundee, Dundee, UK*
[c] *BHF Glasgow Cardiovascular Research Centre, Glasgow, UK*

### 1. Background

For individuals with type 1 diabetes (T1D), minimizing exposure to hyperglycaemia (high glucose), is key in preventing microvascular complications, such as retinopathy, neuropathy and nephropathy. Chronic hyperglycaemia may also contribute to the development of cognitive deficit as reported in groups with T1D when compared to healthy controls [1]. However, some researchers maintain that it is the development of the microvascular complications associated with chronic hyperglycaemia that is the primary contributor to these areas of cognitive deficit [2].

### 2. Purpose

The purpose of this study is to determine whether chronic hyperglycaemia may have an independent effect on cognitive function in adults with T1D. We present the results from our preliminary analysis of a cohort of subjects. This presentation focuses on an estimate of lifetime cognitive change, mean HbA1c percentage, as an index of exposure to chronic hyperglycaemia and severity of retinopathy as a measure of microvascular complication. Our overall hypothesis was that chronic hyperglycaemia, as indicated by high mean HbA1c, will be related to greater decline in cognitive function and that mean HbA1c will have a stronger relationship with cognitive change than recent or current measures of HbA1c. Secondly, chronic hyperglycaemia will have an independent effect on cognitive decline in addition to severity of microvascular complication.

### 3. Methods

Participants include 75 adults aged 45 years or more living in Tayside, Scotland with T1D for 10 years or more. Participants completed neuropsychological assessments in conjunction with regular secondary care review. Measures of premorbid IQ (National Adult Reading Test) and current IQ (Raven's Standard Progressive Matrices) were used in a regression analysis to provide a measure of lifetime cognitive change validated previously on a Scottish cohort [3]. These standardized residual scores were significantly correlated with age ($r = 0.51$, $p < 0.001$). A further regression with age provided an age-adjusted lifetime cognitive change score (ALCC). The ALCC standardized residuals were not correlated with age ($r = 0.00$, $p = 1.00$), premorbid IQ ($r = 0.11$, $p = 0.34$) or years of education ($r = 0.07$, $p = 0.57$) in this cohort.

*Corresponding author. E-mail: hnj2@st-andrews.ac.uk.

ISSN 0953-4180/10/$27.50 © 2010 – IOS Press and the authors. All rights reserved

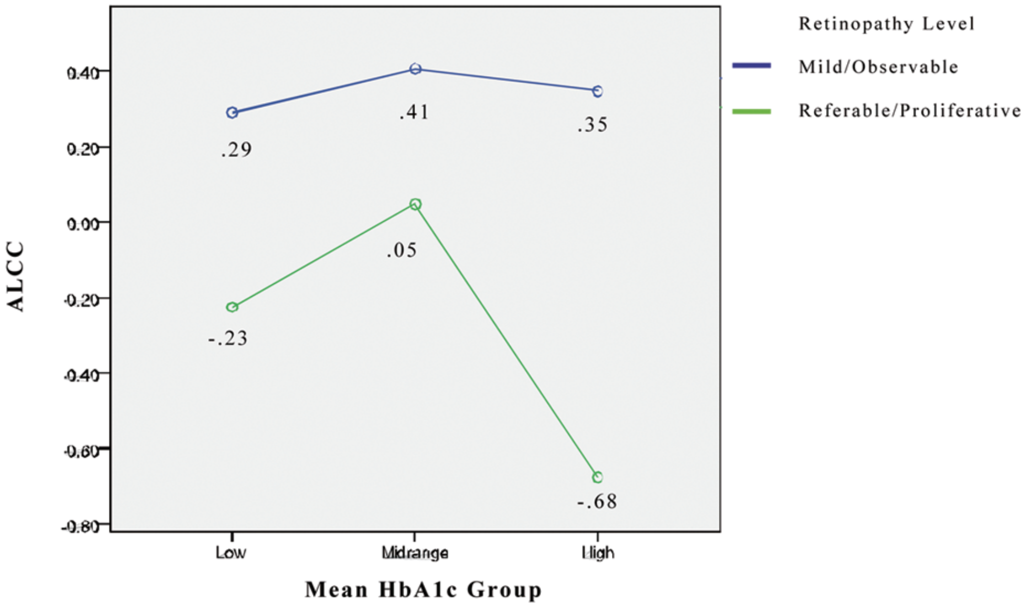

Fig. 1. Factorial ANOVA Age-Adjusted Lifetime Cognitive Change (ALCC) by Mean HbA1c and Retinopathy Level.

Information on HbA1c and retinopathy was obtained with patient consent from the clinical management system used across Scotland for patients with diabetes, Scottish Care Information–Diabetes Collaboration (SCI-DC). A weight was given for each value based on days between readings. In this way, a weighted mean HbA1c was computed using 10 years or more of HbA1c values. In addition, a measure of "Short-Term" HbA1c (weighted mean of previous 5 years), and "Current" HbA1c (HbA1c value on day of testing) was calculated based on previous studies [4,5]. Severity of retinopathy was defined as "the most severe rating on record". Ratings of retinopathy included "Mild", "Observable", "Referable" and "Proliferative" according to the Scottish Diabetic Retinopathy Grading Scheme [6].

### 4. Results

Participants were divided into three groups based on the weighted mean HbA1c score: "Low" ($\leqslant$ 7.9%, $n =$ 22), "Midrange" (8.0 to 8.8%, $n$ = 30) and "High" ($\geqslant$ 8.9%, $n$ = 23) to approximate cut-off points in a previous study using a long term measure of mean HbA1c [7] and to balance group numbers. Results of ANOVA indicated there was no overall difference between groups on the ALCC score, (F(2,72) = 2.48, $p$ = 0.09), however planned comparison showed that the "High" HbA1c group showed a lower mean ALCC score than the "Midrange" group (t(72) = 2.21, $p <$ *0.05*, one-tailed), but not the "Low' group (t(72) = 1.38, $p$ = 0.09, one-tailed). The means for both the "Low" ($\bar{x} = 0.05$) and "Midrange" group ($\bar{x} = 0.24$) are above 0, while the mean for the "High" group ($\bar{x} = -0.36$) is below 0, indicating cognitive decline. There was no significant difference between groups on ALCC when formed based on "Short-Term" HbA1c (F(2,72) = 1.02, $p$ = 0.37) or "Current" HbA1c values (F(2,72) = 0.086, $p$ = 0.92). Mean HbA1c has a slightly stronger, non-significant correlation with ALCC ($r = -$ 0.14, $p$ = 0.12), in comparison to "Short-Term" HbA1c ($r = -$ 0.13, $p$ = 0.13) and "Current" HbA1c ($r = -$ 0.03, $p$ = 0.39).

When retinopathy level and mean HbA1c were both entered into a factorial ANOVA, there was a main effect of retinopathy level (F(1,69) = 7.42, $p <$ 0.01). The overall interaction was not significant (F(2,69) = 0.723, $p$ = 0.49), however planned comparison indicated those with both "High" mean HbA1c and "Referable/Proliferative" retinopathy showed a lower ALCC score than those with "High" mean HbA1c and "Mild/Observable" retinopathy (F(1,71) = 4.81, $p <$ 0.05) as shown in Fig. 1.

### 5. Conclusion

High long-term mean HbA1c is associated with a greater level of cognitive decline than more moderate levels of mean HbA1c. A long-term measure of

HbA1c appears to be a more meaningful indicator of glycaemic control than more limited sampling of Hba1c values based on its stronger relationship with a measure of lifetime cognitive change. Individuals with both high long-term mean HbA1c and proliferative levels of retinopathy may be at most risk for cognitive decline. Preliminary results suggest that chronic hyperglycaemia does not have an independent effect on cognitive change, however may moderate the relationship between retinopathy and cognitive change. Small numbers limit the conclusions that can be drawn from these preliminary results.

### References

[1] A.M.A. Brands, G.J. Biessels, E.H.F. de Haan, L.J. Kappelle and R.P.C. Kessels, The effects of type 1 diabetes on cognitive performance: A meta-analysis, *Diabetes Care* **28** (2005), 726–735.

[2] A.M. Wessels, P. Scheltens, F. Barkhof and R.J. Heine, Hyperglycaemia as a determinant of cognitive decline in patients with type 1 diabetes, *European Journal of Pharmacology* **585** (2008), 88–96.

[3] I.J. Deary, L.J. Whalley and J.R. Crawford, An 'instantaneous' estimate of a lifetime's cognitive change, *Intelligence* **32** (2004), 113–119.

[4] A.M.A. Brands, R.P.C. Kessels, R.P.L.M., Hoogma, J.M.L. Henselmans, J.W. van der Beek Boter, L.J. Kappelle, E.H.F. de Haan and G.J. Biessels, Brain magnetic resonance imaging in older patients with type 1 diabetes, *Diabetes* **55** (2006), 1800–1806.

[5] T. Brismar, L. Maurex, G. Cooray, L. Juntti-Berggren, P. Lindström, K. Ekberg, N. Adner and S. Andersson, Predictors of cognitive impairment in type 1 diabetes, *Psychoneuroendocrinology* **32** (2007), 1041–1051.

[6] Scottish Diabetic Retinopathy Grading Scheme 2007 v1.1, www.ndrs.scot.nhs.uk/ClinGrp/Docs/Grading%20Scheme%202007%20v1.1.pdf.

[7] A.M. Jacobson, G. Musen, C.M. Ryan, N. Silvers, P. Cleary, B. Waberski, A. Vurwood, K. Weinger, M. Bayless, W. Dahms and J. Harth, Long-term effect of diabetes and its treatment on cognitive function, *The New England Journal of Medicine* **356** (2007), 1842–1852.
